# Expression Profile of Genes during Resistance Reversal in a Temephos Selected Strain of the Dengue Vector, *Aedes aegypti*

**DOI:** 10.1371/journal.pone.0039439

**Published:** 2012-08-01

**Authors:** Clare Strode, Maria de Melo-Santos, Tereza Magalhães, Ana Araújo, Contancia Ayres

**Affiliations:** 1 Vector Group, Liverpool School of Tropical Medicine, Liverpool, United Kingdom; 2 Departamento de Entomologia, Centro de Pesquisas Aggeu Magalhães/FIOCRUZ, s/n Cidade Universitária, Recife-PE, Brazil; University of Crete, Greece

## Abstract

**Background:**

The mosquito *Aedes aegypti* is one of the most important disease vectors because it transmits two major arboviruses, dengue and yellow fever, which cause significant global morbidity and mortality. Chemical insecticides form the cornerstone of vector control. The organophosphate temephos a larvicide recommended by WHO for controlling *Ae. aegypti*, however, resistance to this compound has been reported in many countries, including Brazil.

**Methodology/Principal Findings:**

The aim of this study was to identify genes implicated in metabolic resistance in an *Ae. aegypti* temephos resistant strain, named RecR, through microarray analysis. We utilized a custom ‘*Ae. aegypti detox* chip’ and validated microarray data through RT-PCR comparing susceptible and resistant individuals. In addition, we analyzed gene expression in 4^th^ instar larvae from a reversed susceptible strain (RecRev), exposed and unexposed to temephos. The results obtained revealed a set of 13 and 6 genes significantly over expressed in resistant adult mosquitoes and larvae, respectively. One of these genes, the cytochrome P450 *CYP6N12*, was up-regulated in both stages. RT-PCR confirmed the microarray results and, additionally, showed no difference in gene expression between temephos exposed and unexposed RecRev mosquitoes. This suggested that the differences in the transcript profiles among the strains are heritable due to a selection process and are not caused by immediate insecticide exposure. Reversal of temephos resistance was demonstrated and, importantly, there was a positive correlation between a decrease in the resistance ratio and an accompanying decrease in the expression levels of previously over expressed genes. Some of the genes identified here have also been implicated in metabolic resistance in other mosquito species and insecticide resistant populations of *Ae. aegypti*.

**Conclusions/Significance:**

The identification of gene expression signatures associated to insecticide resistance and their suppression could greatly aid the development of improved strategies of vector control.

## Introduction

The genes encoding detoxification enzymes belong to supergene families which have evolved predominantly by gene duplication and functional diversification. The major families are carboxyl esterases, glutathione s-transferase (GSTs) and the monooxygenases P450s. These enzymes can metabolize both endogenous compounds, which are produced by metabolism, and exogenous compounds present in environment, such as insecticides.

Resistance to chemical insecticides as a consequence of increased metabolic capability of these enzymes is known as metabolic resistance, as the insecticide is metabolized or sequestered before reaching its target. In the last few years, many studies have demonstrated the molecular basis of metabolic resistance, and mechanisms such as co-amplification of genes [1], transposon-mediated mutations [2], gene duplication [3] and mutations in trans-regulatory elements have been reported [4]. Li *et al.* [5] have published a good review on this subject.

One of the major threats to the control programmes of vector borne diseases is insecticide resistance, as most of the implemented strategies are based on the exclusive use of such compounds. Thus, managing resistance is fundamental to sustain these strategies.

The design of molecular tools for screening alleles associated with target-site insensitivity in natural populations is feasible, because the molecules involved in this type of resistance are components of nervous system and thus are conserved across different *taxa*, allowing the detection of the same mutation in different species [6]. On the other hand, as mentioned above, the nature of mutations in metabolic genes leading to resistance is diverse and, the development of molecular tools (such as allele specific PCR and TaqMan) that could be used in a wide range of species to detect resistance alleles is a difficult task. Moreover, the amount of genes potentially involved in the metabolism of xenobiotics (more than 200, compared to the very few associated with target-site resistance [6]) makes the task extremely challenging.

The use of microarray analysis to measure and compare gene expression levels between resistant and susceptible mosquito strains has allowed the identification of genes that are involved in specific metabolic resistance mechanisms in *Anopheles gambiae* ([7]; [8] and [9]), *Anopheles arabiensis* [10] and in *Ae. aegypti* [11]. David *et al.* [12] have constructed a microarray containing more than 200 detoxification gene specific for *An. gambiae* and have used this chip to investigate metabolic-based insecticide resistance. Similarly, Strode *et al.* [13] developed the *Ae. aegypti* ‘Detox Chip’, which also contains more than 200 genes putatively involved with metabolic resistance. This Detox Chip has also been used to evaluate mosquito response to xenobiotic exposure ([14]; [15] and [16]). These arrays represent today the only available tool to rapidly identify genes involved in metabolic resistance and may provide valuable information for resistance management.

Almost half of the world's population (2.5 billion) are believed to be at risk from Dengue [17] and this is due in no small part to the fact that the vector *Ae. aegypti* is a mosquito which has superbly adapted to human activity and increasing urbanization. With no available vaccine, vector control is the only option in the fight against Dengue and insecticides are a vital weapon. Temephos is an organophosphate larvicide recommended by WHO to control *Ae. aegypti* larvae and is even sanctioned for use in potable water containers. This insecticide has been used intensively in Brazil to control *Ae. aegypti* since the 1990′s as an exclusive larvicide, with no alternative compounds ever being used [18]. Moreover, during critical outbreaks its use has been intensified. Hence, an alteration in the susceptibility status of *Ae. aegypti* has been reported in many localities from Brazil ([18]; [19] and [20]).

The National Programme for Dengue Control (PNCD) has recommended temephos substitution by biological larvicides, such as *Bacillus thuringiensis israelensis* (Bti) or insect growth regulators, like diflubenzuron, a chitin synthesis inhibitor, in areas where temephos resistance has been detected. The aim is to manage resistance by stopping the use of temephos in order to allow the resistant population to revert to susceptible [21] after which time temephos could be reinstated. However the time it takes for the reversal of resistance to this compound in *Ae. aegypti* populations is very slow [20].

In order to study the progress and reversal of temephos resistance in *Ae. aegypti*, Melo Santos *et al.* [22] developed a strain (RecR) with a high resistance level (RR = 180). In addition, they have simulated three different field conditions to observe resistance reversal that involved cessation of temephos exposure and/or the introduction of susceptible mosquitoes into the resistant colony. The present study aimed to identify individual genes associated with resistance in RecR, and to evaluate the gene expression profiles before and after the reversion of resistance.

## Results

### Bioassay results

Following 20 generational selections with temephos, the RecR strain had achieved a RR_50_ and RR_90_ of 175 and 181 respectively, when compared with the susceptible Rockefeller strain (Table 1). When temephos selection was stopped after 13 generations, the RR dropped dramatically to 4.4 (RR_50_) and 6.5 (RR_90_).

**Table 1:** Values of lethal concentration (LC) of temephos and resistance ratios (RR) for the *Ae. aegypti* strains.

Strain	Number of exposed larvae	LC_50_ mg/L (Fiducial limits)	LC_90_ mg/L (Fiducial limits)	RR_50_	RR_90_
RecR _F20_	1380	1.23 (1.16–1.3)	1.81 (1.69–1.97)	175	181
RecRev1_F13_	1200	0.031 (0.022–0.045)	0.065 (0.056–0.078)	4.4	6.5
RecL	1440	0.009 (0.008–0.010	0.015 (0.013–0.016)	1.3	1.5
Rockefeller	2240	0.007 (0.006–0.008	0.010 (0.010–0.011)	1	1

RecR =  Brazilian resistant strain; RecRev1_F13_ =  sub colony of the RecR without temephos exposure; Rockefeller: susceptible laboratory strain; RecL: Brazilian susceptible laboratory strain.

### Biochemical assays

Biochemical analysis showed no significant alteration in enzyme activity when RecL and RecRev1 were compared to the to the Rockefeller strain (Table 2). On the other hand, RecR displayed alterations of α-esterases, GSTs and P450s, in comparison to Rockefeller. The decrease in α-esterases and GST activities in the F20 downgraded the population's resistance status from highly altered (HA) to altered (A).

**Table 2:** Enzymatic activity associated with esterases (α, β and PNPA) acetylcholinesterase (ACE), glutathione-S-transferases (GST) and mixed function oxidases (MFO) observed in *Ae. aegypti* strains susceptible and resistant to temephos.

Enzyme assayed	*Ae. aegypti* strain	No. of females	p99^ e^	N > p99^ f^	% > P99^ g^	Status^h^
**ACE** (**% act**)	Rockefeller a	106	29.71			
	RecR **^b^**_F20_	116		2	2%	U
	RecRev1 **^c^**_ F13_	102		0	0	U
	RecL **^d^**	98		0	0	U
**GST**(**mmol/mg ptn/min**)	Rockefeller	114	1.97			
	RecR _F20_	115		30	26%	A
	RecRev1_F13_	73		4	5%	U
	RecL	69		3	4%	U
**MFO** (**P450**)(**nmoles cit/mg ptn**)	Rockefeller	113	47.35			
	RecR _F20_	117		40	34%	A
	RecRev1_F13_	106		2	2%	U
	RecL	103		8	8%	U
**α-esterase(nmol/mg ptn/min)**	Rockefeller	104	40.87			
	RecR _F20_	116		55	47%	A
	RecRev1_F13_	108		2	2%	U
	RecL	74		8	11%	U
**β-esterase**(**nmol/ mg ptn/min**)	Rockefeller	112	71.46			
	RecR _F20_	113		5	4%	U
	RecRev1_F13_	108		0	0%	U
	RecL	120		4	3%	U
**Esterases-PNPA**(**Δabs/mg ptn/ min**)	Rockefeller	119	2.96			
	RecR _F20_	100		6	6%	U
	RecRev1_F13_	NT		NT	NT	NT
	RecL	120		1	1%	U

a Rockefeller: susceptible laboratory strain; ^b^ RecR =  Brazilian resistant strain; ^c^ RecRev1_F13_ =  sub colony of the RecR without temephos exposure; ^d^ RecL: Brazilian susceptible laboratory strain.^ e^ 99 percentile for Rockefeller. ^f^ Number of RecR individuals with 99 percentile above than the 99 percentile for Rockefeller. ^g^ Percentage of individuals with 99 percentile above than the 99 percentile for Rockefeller. ^h^ Classification of enzyme activity compared to control (Rockefeller): unaltered (U); altered (A) and highly altered (HA). NT  =  not tested.

### Microarrays

**Figure 1:**
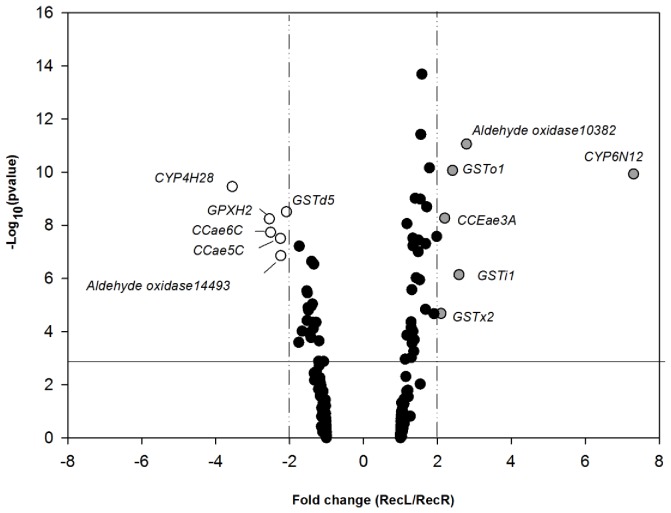
Differential expression of *Ae. aegypti* detoxification genes in larvae of the parental RecL and RecR strains. Differences are indicated as a function of both expression ratio (X-axis) and significance, expressed as the negative log10 scale of the p-value of the t-test of the fold change between the groups (Y-axis). Vertical lines indicate two-fold expression differences in either direction. The horizontal line indicates the significance threshold of P<0.001 adopted for the one sample t-test.

A comparison on the transcription profiles of the RecL and RecR 4th instar larvae identified a total of 12 significantly differentially expressed genes (Fig. 1). Six genes were over expressed in the RecR strain (Table 3); a single P450 (*CYP6N12*), three GSTs (*GSTi1, GSTo1* and *GSTx2*), one COE (*CCEae3A*) and a peroxinectin (*Aldehyde oxidase 10982*). At 7.03 fold *CYP6N12* demonstrated the highest level of over expression.

**Table 3:** Microarray results of over expressed genes in the 4^th^ instar larvae of the RecR strain when compared with larvae from the parental RecL strain.

Class of gene	Gene ID (vectorbase)	Gene name	Fold change	−Log_10_ (p value)
P450	AAEL009124	*CYP6N12*	7.03	9.92
Peroxinectin	AAEL010382	*Aldehyde oxidase10382*	2.79	11.05
GST	-	*GSTo1*	2.41	10.06
Carboxylesterase	AAEL005112	*CCEae3A*	2.2	8.27
GST	AAEL010500	*GSTx2*	2.1	4.7

**Figure 2:**
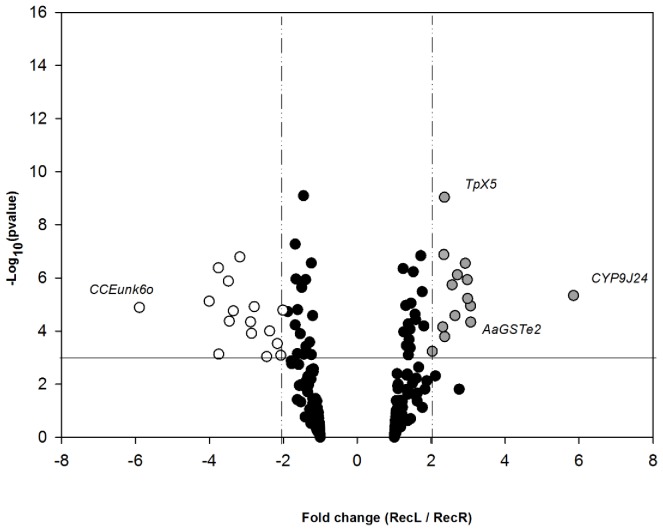
Differential expression of *Ae. aegypti* detoxification genes in 3 day old female mosquitoes of the parental RecL and RecR strains. Differences are indicated as a function of both expression ratio (X-axis) and significance, expressed as the negative log10 scale of the p-value of the t-test of the fold change between the groups (Y-axis). Vertical lines indicate two-fold expression differences in either direction. The horizontal line indicates the significance threshold of P<0.001 adopted for the one sample t-test.

In the case of 3 day old females, a larger number of genes, 29, were significantly differentially expressed between the RecL and RecR strains (Fig. 2). Of the 13 genes that were over expressed in the RecR strain (Table 4), 8 were P450s (*CYP9J24, CYP9J32, CYP4H28, CYP6AG7, CYP6CB2, CYP6N12, CYP9M9* and *CYP9J10*), two GSTs (*GSTe2* and *GSTe3*), two aldo-keto reductases (*4118* and *15002*), and a single thioredoxin peroxidise (*TpX5*). The P450 *CYP9J24* showed the greatest over expression with a 5.85 fold change.

**Table 4:** Microarray results of over expressed genes in 3 day old females of the RecR strain when compared with females from the parental RecL strain.

Class of gene	Gene ID (vectorbase)	Gene name	Fold change	−Log_10_(p value)
P450	AAEL014613	*CYP9J24*	5.85	5.34
GST	AAEL007951	*GSTe2*	3.1	4.34
Aldo-keto reductase	AAEL004118	*Aldo-keto reductase4118*	3	5.23
GST	AAEL007947	*GSTe3*	3	5.94
P450	AAEL008638	*CYP9J32*	2.92	6.56
P450	AAEL003380	*CYP4H28*	2.71	6.12
P450	AAEL006989	*CYP6AG7*	2.64	4.58
Aldo-keto reductase	AAEL015002	*Aldo-keto reductase15002*	2.57	5.74
P450	AAEL002872	*CYP6CB2*	2.36	3.8
Thioredoxin peroxidase	AAEL009051	*TpX5*	2.36	9.03
P450	AAEL009124	*CYP6N12*	2.34	6.88
P450	AAEL001807	*CYP9M9*	2.31	4.16
P450	AAEL014614-RA	*CYP9J10*	2.12	2.31

### Expression levels of *CYP6N12,* Aldehyde oxidase 10382, *GSTi1*, *GSTo1*, *GSTx2*, and *CCEae3A*

**Figure 3:**
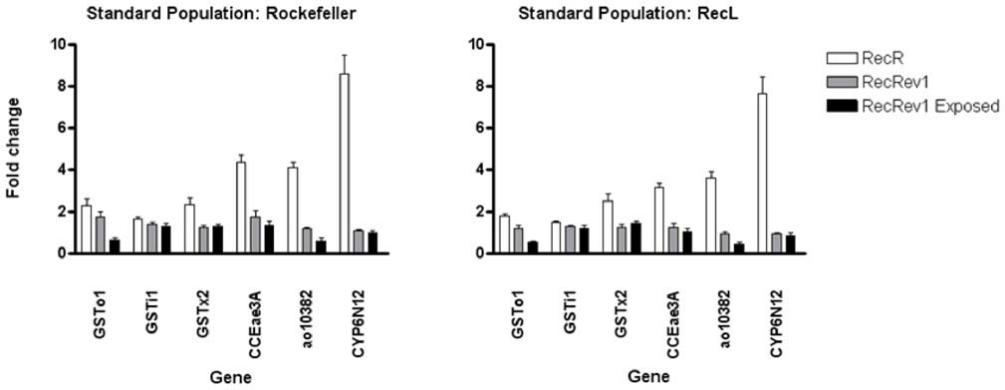
Fold-change in *CYP6N12, Aldehyde oxidase 10382* (*ao10382*), *GSTi1, GSTo1, GSTx2*, and *CCEae3A* transcripts in RecR, RecRev1 and RecRev1 Exposed strains, compared to Rockefeller or RecL strains.

Real-time PCR (RT-PCR) was used to validate the differentially expressed genes in RecR mosquitoes detected by microarrays. According to RT-PCR results and using the expression levels of either the Rockefeller or RecL strains as the baseline, the expression of *CYP6N12*, Aldehyde oxidase 10382, *GSTi1*, *GSTo1*, *GSTx2*, and *CCEae3A* transcripts were, in general, higher in the RecR strain than in RecRev1 and RecRev1 Exposed mosquitoes (Fig. 3). RecRev1 and RecRev 1 Exposed showed similar expression levels for all of the genes tested, although values were slightly lower in RecRev1 Exposed, except for *GSTx2*. The sharpest increase in the amount of transcripts found in RecR compared to the other strains was with *CYP6N12* where it was 8.6 (SD±1.8) and 7.6 (SD±1.5) fold higher compared with Rockefeller and RecL respectively.

## Discussion

The decision to use the RecL as the baseline strain to analyze gene expression in the resistant strain RecR, rather than the Rockefeller strain, was taken in order to minimize biases linked to natural variations among populations or to long term lab colonization. This ensured a closer genetic match between the susceptible and resistant mosquitoes which would give us more confidence that any genes that we subsequently found to be over expressed in RecR were indeed linked to insecticide resistance. The degree of resistance between the two strains was RR_90_ of 7.0 [22] at the beginning of the resistance selection process.

Insensitive acetylcholinesterase (iAChE) is not believed to be a contributing factor in RecR Temephos resistance [22]. Biochemical assays performed on RecR suggested the involvement of metabolic resistance and that GST and COE-based activity were driving resistance. This finding is supported here by the microarray and RT-PCR data, which identified the involvement of *GSTi1, GSTo1, GSTx2* and *CCae3A*. The biochemical tests suggested a negligible role for P450s in larvae, however in this study the gene with the strongest over expression was the P450 *CYP6N12*. Biochemical assays lack specificity and sensitivity and do not offer the resolution offered by microarrays. Molecular assays may indicate the involvement of molecules that are missed in the currently available biochemical tests whilst biochemical assays may reflect changes in enzyme activity/specificity which in turn are obviously undetectable by microarrays, so both assays are complementary.

Despite the fact that only the larvae were exposed to temephos, we observed a higher number of over expressed genes in adult females, the majority of which were P450s. The obvious explanation would be that the population has cross resistance to other insecticides that are applied as adulticides, but this is not the case for this strain, which is susceptible to the pyrethroids deltamethrin, cypermethrin, malathion (organophosphate) and pyriproxyfen (a juvenile hormone analogue) [22]. Only one gene, *CYP6N12*, was over expressed in both larvae and adult females, albeit at lower levels in the latter. The application of temephos against the larval stage is likely to be selecting for the over expression of *CYP6N12*, and although this molecule may offer residual protection in adults, it is possible that expression gradually diminishes in adults as temephos exposure recedes. *CYP4H28* expression was also altered in both life stages but demonstrated a contradictory pattern as it was significantly under expressed in RecR larvae and over expressed in adult females. It is difficult at this stage to make any inferences about the possible role of this particular gene. A number of physiological processes performed by female mosquitoes, such as host seeking, blood feeding and reproduction, may affect the expression of metabolic genes [23], [24]. However, the current study was performed with non-blood fed 3-day old females of RecL and RecR populations, ensuring that the differences observed are the result of resistance rather than to other physiological processes.

Molecular assays such as microarrays have helped expand the number of resistance populations screened for genes putatively conferring resistance, so it will be interesting to see whether gene signatures are insecticide specific and/or geographically biased. With the exception of *GSTo1*, all of the over expressed genes observed in the RecR strain have been identified in other mosquito populations. Over expression of *CYP6N12* has been demonstrated in larvae of susceptible *Ae. aegypti* Bora Bora strain when they were exposed to either sub lethal doses of permethrin, the heavy metal copper, the polycyclic aromatic hydrocarbon (PAH) fluoranthene [25], [26] and the herbicide glyphosate [27]. *GSTx2* over expression was recorded in Bora Bora larvae exposed to the carbamate insecticide propoxur [28], whilst propoxur, glyphosate and benzo [a] pyrene [27] induce the expression of *GSTi1.* While the expression of the above P450s and GSTs were induced in the Bora Bora strain, elevated *CCEae3A* expression was seen in a temephos- and deltamethrin-resistant population from Martinique in the West Indies [11].

The cohort of up-regulated genes observed in RecR adults (*CYP9J32, CYP9J24, CYP9J10, GSTe2* and *GSTe3*) were also over expressed in an *Ae. aegypti* permethrin resistant strain from Isla Mujeras in Mexico [13]. In the same study, *CYP9J32* was also up-regulated in a DDT and pyrethroid resistant population from Thailand and in a deltamethrin resistant Vietnamese strain [29]. The crucial question is whether genes identified in microarray studies encode proteins with insecticide metabolizing properties and thus are functionally associated with resistance. In the case of *CYP9J32, CYP9J10, CYP9J24* and *GSTe2* the answer is yes. All three P450s have deltamethrin metabolizing activity *in vitro* but whilst *CYP9J10* and *CYP9J24* can also metabolise permethrin this is not the case for *CYP9J32* [30]. The fact that some P450s appear to be broad acting whilst others appear to be more specific even within the same insecticide class has also been observed with *CYP6AA3* and *CYP6P7* from *An. minimus* [31] and highlights the complexities involved in metabolic resistance. Recombinant GSTe2 has been show to be involved in the dehydrochlorination of DDT in both *Ae. aegypti* [32] and *An. gambiae* [33] and partial silencing of *GSTe2* in *Ae. aegypti* leads to a reduction of resistance in the mosquito [34]. What we don't know at this stage is whether the over expressed genes observed in this study are similarly effective against temephos. This issue is currently being addressed at LSTM alongside the profiling of *CYP6N12* enzymatic activity against a range of insecticides.

One of the presumptions of resistance management, is that by ceasing the selection pressure on an insect population, the resistant phenotype will most probably revert to susceptible [35]. This strategy is based on evidence which suggests resistance is associated with fitness costs. For example, pyriproxyfen resistance in *Bemisia tabaci* resulted in a reduction of 25% in life characteristics such as nymph survival, sex ratio, fecundity, egg hatching rate and development time [36]. Halting a spraying campaign once resistance has been detected or alternating the use of different classes of insecticides are recommended tactics to control the spread of insecticide resistance. Few studies have investigated the reversal of resistance and none have demonstrated a decrease in the expression of genes implicated in conferring resistance.

One of the most interesting findings of the current study was that a decrease in temephos resistance was followed by decreases in expression levels of the previously significantly over expressed genes and a reduction in metabolic enzyme activities. When temephos exposure was terminated at F13, there was an increase in temephos susceptibility in RecRev1 after 13 generations. With the exception of *GSTi1*, all of the analyzed RecR over expressed genes presented lower expression in the RecRev1 population. The most significant decline was observed with *CYP6N12*, which dropped from ∼7.5 fold to ∼1 fold. This finding reinforces the assumption that these genes are directly involved in conferring temephos resistance in this strain.

The speed with which over expression of metabolic enzymes become fixed in a population is not clear and one intriguing question is whether metabolic genes are induced following insecticide application or whether they are constitutively expressed. In order to determine whether a single exposure to temephos induced gene expression, the RecRev1 population was subjected to insecticide exposure. Across the six genes analysed, there was no significant over expression upon temephos exposure in RecRev1 individuals. In fact, *Aldehyde oxidase 10382* and *GSTo1* demonstrated a ∼50% reduction in fold change expression. The reason for such a dramatic reduction in the expression of these two genes is unclear.

Induction of gene expression has been observed in susceptible strains of *Ae. aegypti* after exposure of sub-lethal doses of insecticides or xenobiotics [15], [16], [28]. Although the constitutive expression of genes that encode for detoxifying enzymes may appear more costly than inducing them following exposure, it ensures that the mosquito is always primed for an insecticide “attack”. This strategy may be more crucial for insects that are constantly under selection pressure in the wild. Once the chemical threat has diminished, gene expression may then subside over time, which appears to be the case in this study. How quickly this can occur in nature is an issue that would require further research. Although there is limited information on this, the dynamics of resistance reversal can vary depending on the insecticide being used. For example, persistence of resistance following the withdrawal of an indoor residual spraying (IRS) campaign, in *An. culicifacies* to DDT, malathion and deltamethrin took 30, 9 and 2–3 years, respectively in mosquito populations from India [37]. Determining the characteristics of induction/reversal of resistance would ultimately empower decision making in vector control programmes.

Aside from P450, GST and COE over expression we also observed elevated levels of genes such as a peroxinectin, aldo-keto reducatases and thioredoxin peroxidase. Oxidative stress response genes have been previously observed in insecticide resistant mosquitoes ([9], [10], [13], [15] and [16]), which suggests that resistant strains are under a higher oxidative stress condition, which may be linked to the fitness costs associated with resistance.

It is becoming increasingly clear that gene signatures are appearing across different insecticide resistant populations of *Ae. aegypti* from around the world which are not necessarily exclusive to a particular chemical. Theses signatures will hopefully assist in the development of diagnostic tools for metabolic resistance. The availability of whole genome arrays for *Ae. aegypti* will allow us to begin the process of examining the insecticide resistant strains on a wider scale which could lead us to identifying genes and pathways aside from metabolic resistance that contribute to the phenotype. A clear understanding of the genetic factors underpinning resistance and their potential suppression is vital for the development of vector control programmes.

## Materials and Methods

### Mosquito strains

In the present study, four mosquito strains were used. *Ae. aegypti* RecR, a temephos resistant strain previously developed by Melo-Santos *et al.* [22] was used at F_20_, in the microarray and q-RT-PCR assays,. This strain has been previously tested against other chemical insecticides and showed to be susceptible to malathion (organophosphate), cypermethrin, deltamethrin (pyrethroid) and pyriproxyfen (a juvenile hormone analogue). *Ae. aegypti* RecL was used as a reference of susceptibility to temephos. This strain is original from Recife, Pernambuco, Brazil and has been kept in the Department's insectary at CPqAM for over 15 years [38]. *Ae. aegypti* Rockefeller was also used as a standard susceptible strain in the RT-PCR experiments only. RecRev1 strain was used in the RT-PCR tests. This is a sub colony from RecR that has been maintained for 13 generations without temephos exposure and thus, its resistance ratio has dropped to a lower level [22]. Table 1 shows the resistance ratio for each strain.

### Biochemical assays

Biochemical tests were performed with female according to the protocol recommended by The Ministry of Health [39] *et al.* aiming to verify the activity of esterases (using α and β naphthyl as substrates for α-Est and β-Est, respectively, and p-nitrophenyl PNPA), mixed function oxidases (MFO) or P450s, glutathione S-transferases (GST), and acetylcholinesterase (ACE). Analysis of biochemical data were performed using *GEN 5* software, which classifies populations as unaltered (≤15%), altered (>15% <50%) and highly altered (>50%), based on the percentage of individuals from each population with enzymatic activity above the Rockefeller 99th percentile.

### Microarrays

#### Total RNA extractions

RNA extractions, cDNA synthesis and labeling reactions were performed independently for each biological replicate. Total RNA was extracted from pools of 30 4^th^ instar larvae or pools of 30 non-blood fed 3 day old female mosquitoes using a PicoPure™ RNA isolation kit (Arcturus) according to manufacturer's instructions. Total RNA quantity and quality were assessed using Nanodrop spectrophotometer (Nanodrop Technologies, UK) and gel electrophoresis before further use.

#### Direct labelling and hybridizations

RNA was amplified using a RiboAmp™ RNA amplification kit (Arcturus) according to the manufacturer's instructions. Amplified RNAs were checked for quantity and quality by spectrophotometry and bionalayser (Agilent). Amplified RNA was reverse transcribed into labelled cDNA and hybridised to the array as previously described [13]. Labeled cDNAs were hybridized to the *Ae. aegypti* ‘detox chip’ (LIV *Ae. aegypti* DETOX 0.3K v2, Vectorbase) Each RecL vs RecR comparison was repeated three times with different biological samples. For each biological replicate, two hybridizations were performed in which the Cy3 and Cy5 labels were swapped between samples, hence a total of six hybridisations were performed for each comparison.

### Data Analysis

Microarray spots that failed to meet any of the following criteria in either channel were rejected as poor quality and eliminated from subsequent analysis; (i) an intensity value of >300, (ii) signal-to-noise ratio of >3 and (iii) greater than 60% of pixel intensity superior to the median of the local background ± 2 SD. Normalisation and statistical analyses of the data was performed using the Limma 1.9 software package for R 2.3.1, available from the CRAN repository (http://www.r-project.org). Background corrected intensities from the red, (*R*, Cy5), and the green, (*G,* Cy3), channel were transformed to intensity log-ratios, *M* = logR/G, and their corresponding geometrical means, *A* = (logR + log G)/2. Within each array *M*-values were normalized as a function of *A* using the *Lowess* [40] scatter plot smoothing function and scaled to equalize the median absolute value across all arrays to account for technical biases between replicate hybridisations.

Mean expression ratios were submitted to a one-sample student's t-test against the baseline value of 1 with a multiple testing correction (Benjamini& Hochberg false discovery rate). Genes showing both a *t-*test and p-values of <0.001 and ±2 fold expression were considered to be differentially expressed. Expression data has been deposited and is accessible at Vectorbase (http://www.vectorbase.org/index.php).

### Quantitative real-time PCR (RT-PCR) of genes over-expressed in RecR larvae

These experiments were performed with two aims: to confirm the results obtained from microarrays in regard to genes that are over-expressed in RecR larvae (Table 3), and to check the expression level of these genes in larvae of RecRev1 strain and in RecRev1 individuals that have survived an exposure to temephos (RecRev1 exposed).

Total RNA was extracted from pools containing five L4 from either of the following *Ae. aegypti* strains: 1) Rockefeller; 2) RecL; 3) RecR (F20); 4) RecRev1 (F13); and 5) RecRev1 exposed. A total of 12 pools per population were assayed in RT-PCRs. RNA was extracted with Trizol® Reagent (Invitrogen), by following the manufacturer's protocol, and samples were further treated with DNA-free DNase (Ambion) to ensure total elimination of genomic DNA. Two micrograms of the RNA were then used to synthesize cDNA by utilizing AMV reverse-transcriptase and oligo(dT)_20_ (Invitrogen), according to the manufacturer's instructions. One microliter of the cDNA was used per well in the RT-PCR assays.

Primers that amplify a region of about 100–200 bp of the coding sequence were designed for the selected genes. The *rpl8* gene [41] was used as the endogenous control. RT-PCR primers for *rpl8, CYP6N12, Aldehyde oxidase 10382, GSTi1, GSTo1, GSTx2*, and *CCEae3A* amplified regions of 122, 135, 178, 114, 207, 107, and 128 bp of the transcripts, respectively. The SYBR Green PCR Master Mix (Applied Biosystems) was used for the PCRs, which were performed under the following conditions: 50°C for 2 min, 95°C for 10 min, and 40 cycles of 15 s at 95 C, 30 s at 54°C and 1 min at 72°C. The relative expression of each gene in RecR, RecRev1 and RecRev1 Exposed, compared to Rockefeller or RecL strains, was calculated through the method 2^−ΔΔCT^ [42], as previously described [43].

### Exposure of RecRev1 to temephos

In order to check if the gene expression profile was a consequence of immediate insecticide challenge, 300 RecRev1 larvae were exposed to a single dose of temephos (0.06 mg/L), enough to eliminate 100% of susceptible individuals. The surviving larvae were collected to be analyzed by RT-PCR.
